# Phononic-Crystal-Based SAW Magnetic-Field Sensors

**DOI:** 10.3390/mi14112130

**Published:** 2023-11-20

**Authors:** Mohsen Samadi, Julius Schmalz, Jana Marie Meyer, Fabian Lofink, Martina Gerken

**Affiliations:** 1 Integrated Systems and Photonics, Department of Electrical and Information Engineering, Kiel University, Kaiserstraße 2, 24143 Kiel, Germany; jusc@tf.uni-kiel.de; 2 Fraunhofer Institute for Silicon Technology ISIT, 25524 Itzehoe, Germany; jana.meyer@isit.fraunhofer.de (J.M.M.); fabian.lofink@isit.fraunhofer.de (F.L.); 3 Kiel Nano, Surface and Interface Science (KiNSIS), Kiel University, Kaiserstraße 2, 24143 Kiel, Germany; 4 Microsystem Materials, Department of Materials Science, Kiel University, Kaiserstraße 2, 24143 Kiel, Germany

**Keywords:** magnetic field sensing, phononic crystal, surface acoustic waves, ∆*E* effect

## Abstract

In this theoretical study, we explore the enhancement of sensing capabilities in surface acoustic wave (SAW)-based magnetic field sensors through the integration of engineered phononic crystals (PnCs). We particularly focus on amplifying the interaction between the SAW and magnetostrictive materials within the PnC structure. Through comprehensive simulations, we demonstrate the synchronization between the SAWs generated by IDTs and the resonant modes of PnCs, thereby leading to an enhancement in sensitivity. Furthermore, we investigate the Δ*E* effect, highlighting the sensor’s responsiveness to changes in external magnetic fields, and quantify its magnetic sensitivity through observable changes in the SAW phase velocity leading to phase shifts at the end of the delay line. Notably, our approach yields a magnetic field sensitivity of approximately $S\sim 138\;\frac{{}^{\circ}}{mT}$ for a delay line length of only 77 µm in homogeneous magnetic fields. Our findings underline the potential of PnCs to advance magnetic field sensing. This research offers insights into the integration of engineered materials for improved sensor performance, paving the way for more effective and accurate magnetic field detection solutions.

## 1. Introduction

Magnetic field sensors play a crucial role in various applications, providing an accurate measurement and detection of magnetic fields [1,2,3,4,5,6,7]. The use of surface acoustic wave (SAW) sensors for magnetic field sensing has gained significant attention owing to their high sensitivity and ability to detect small changes in magnetic fields [8,9,10,11]. A specific sensing principle utilized in SAW magnetic field sensors is the change in the Young’s modulus of magnetostrictive materials in the presence of an external magnetic field (∆*E* effect) [12,13,14,15,16]. The magnetically induced change in the Young’s modulus leads to the phase modulation of the propagating acoustic wave, which can be detected and measured, providing information about the magnetic field strength.

Phononic crystals (PnC) [17,18,19,20,21], i.e., engineered materials exhibiting periodic arrangements, allow for the manipulation of the propagation of acoustic waves. By tailoring the periodicity and composition of PnCs, researchers have achieved precise control over acoustic wave propagation and dispersion characteristics [22,23]. The band structure of PnCs can be actively tuned by modifying the elastic properties of the constituent materials via the application of external stimuli. In particular, numerous studies have revealed significant variations in the band structure of PnCs composed of magnetostrictive materials when exposed to a magnetic field [24,25,26,27,28,29,30]. Here, we investigate the integration of accurately designed PnC structures into SAW magnetic field sensors for advancements in sensitivity and overall sensor performance, holding promise for unlocking novel applications in industries where accurate and efficient magnetic field detection is of paramount importance. 

**Figure 1:**
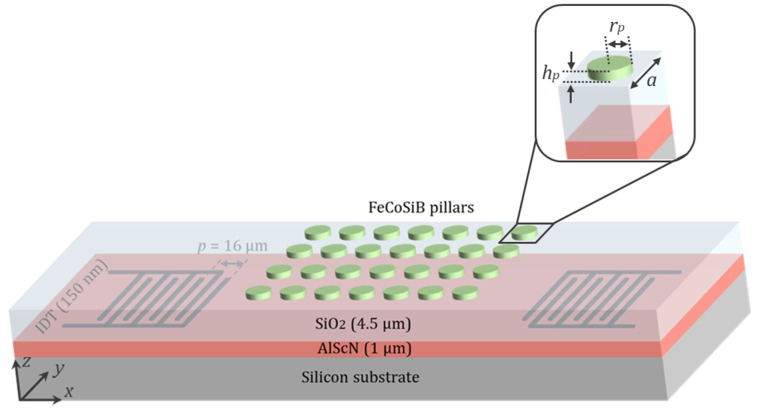
Scheme of the SAW magnetic field sensor with PnC. A square lattice of FeCoSiB pillars is patterned on top of a SiO_2_ layer to build a PnC between the IDTs. A unit cell of the PnC is depicted with finer details in the magnified view. *a* represents the lattice constant, while *r_p_* and *h_p_* are the radius and thickness of the pillars, respectively.

To do so, similar to what proposed in [31], we consider a thick silicon substrate covered by a 1 µm thick AlScN piezoelectric layer [32,33,34]. As depicted in Figure 1, two inter-digital transducers (IDTs) are introduced on the surface of the AlScN layer to stimulate and detect the SAW via the piezoelectric effect. The dimensions of the IDTs are engineered to create and capture a Rayleigh wave at the center frequency of *f*~250 MHz. A PnC structure composed of a 2D square lattice of FeCoSiB pillars is patterned between the two IDTs. A SiO_2_ layer is deposited on top of the piezoelectric layer to confine the acoustic wave close to the surface, thereby boosting the interaction between the SAW and the magnetostrictive material. Moreover, this minimizes the surface roughness of the underlying layer, allowing for an improved quality of the magnetostrictive layer.

The dimensions and arrangement of the pillars are precisely tailored to create a resonant mode coinciding with the frequency at which the Rayleigh wave is initiated and guided through the delay line. This approach ensures that the SAW generated by the IDT efficiently couples with a resonant mode of the PnC. Consequently, this synchronization amplifies the interaction between the SAW and the magnetostrictive material, resulting in an enhanced sensor’s responsiveness to changes in the magnetic field. Here, we employ a finite element analysis to illustrate the practical realization of this concept and elucidate its potential impact on enhancing the sensitivity of magnetic field sensors based on SAW.

Various models have been utilized in prior research to describe the behavior of magnetostrictive materials [26,35,36,37]. In our model, we evaluate the impact of magnetic field strength on the elastic properties of the magnetostrictive material and, as a result, the velocity of SAWs propagating through the delay line. It is essential to note that our model does not account for the direction in which the magnetic field is applied. Additionally, it is worth noting that the shape of the magnetostrictive pillars can introduce magnetic anisotropy, a factor that is not fully addressed in our current model. We recognize that these limitations may potentially impact the accuracy of our predictions, especially when the direction of the applied magnetic field is a crucial factor in the system under investigation. What sets our model apart from previous research is our comprehensive approach. Rather than solely focusing on band structure calculations for a single unit cell of the PnC, we develop a full 3D model of an SAW magnetic field sensor with a delay line configuration and an embedded PnC structure. This model enables us to thoroughly assess the influence of the applied magnetic field strength on the dispersion and propagation characteristics of the SAW as it travels along the delay line.

The paper is structured as follows. In Section 2, the theoretical model is described. In Section 3, the results are presented. This section is divided into subsections that explore the band structure of the PnC, the transmission of SAWs through the PnC, and the use of the Δ*E* effect as a mechanism for detecting magnetic fields. Finally, conclusions are drawn in Section 4.

## 2. Theoretical Model

The sensor’s functionality is characterized by solving a set of coupled differential equations using the finite element method (FEM) [16,38]:

(1) $$\nabla .\sigma =-\rho \omega^{2}u$$

(2) $$\nabla .D=\rho_{c},$$

(3) ∇.B=0,

where σ, ρ, $\rho_{c}$, ω, and u are the stress tensor, the mass density, the free charge density, the angular frequency, and the displacement vector, respectively. **D** and **B** are the electric and magnetic flux density vectors. The material properties are given in Appendix A. The mechanical equation of motion (Equation (1)) is coupled to the electrostatic equation (Equation (2)) and magnetostatic equation (Equation (3)) via the constitutive piezoelectric equations in the stress–charge form:

(4) $$\sigma =C\varepsilon -e^{T}E,$$

(5) $$D=e\varepsilon -\varepsilon_{el}E.$$

Here, ε and C represent the strain tensor and the mechanical stiffness tensor, while the piezoelectric tensor and the electrical permittivity tensor are denoted as e and $\varepsilon_{el}$. At low frequencies, one can neglect the impact of eddy currents and express the electric field **E** as follows [15]:

(6) E=−∇V,

where *V* is the electric potential.

**Figure 2:**
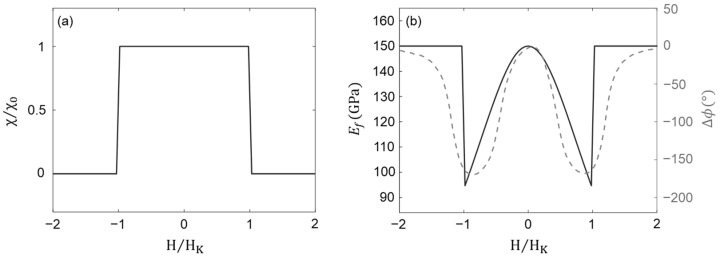
(**a**) Normalized dynamic differential susceptibility $\chi /\chi_{0}$ modeled using the quasi-static solution at low-frequency regime. (**b**) The Young’s modulus of FeCoSiB (solid curve) acquired at different values of applied magnetic field H normalized to the effective anisotropy field $H_{K}$. For comparison, we present the phase shift measurements derived from a SAW delay line comprising a FeCoSiB layer with the same material parameters as those used in our calculations (dashed curve).

In this study, we consider an isotropic magnetostrictive material and analyze its characteristics using Hooke’s law:

(7) σ=Cε,

where the mechanical stiffness tensor C is determined by Young’s modulus (*E*) and Poisson’s ratio (*ν*). Applying an external magnetic field **H** to the magnetostrictive material alters the Young’s modulus to an extent that can be quantified using the following formula [13]:

(8) $$\frac{1}{∆E}=\frac{9}{4}\frac{\mu_{0}\lambda_{s}^{2}H^{2}}{K^{2}}\chi ,$$

where $\mu_{0}$, $\lambda_{s}$, and K are the magnetic vacuum permeability, the saturation magnetostriction, and the first-order anisotropy constant, respectively. Within the frequency range investigated in this research, the differential magnetic susceptibility χ follows the quasi-static approximation, and for $\left|H\right|<H_{K}$, it can be regarded as equivalent to its static value $\chi_{0}$ [13]. Here, $H_{K}$ represents the effective anisotropy field and can be expressed by:

(9) $$H_{K}=\frac{2K}{\mu_{0}M_{s}},$$

with $M_{s}$ denoting the saturation magnetization. The variation in the dynamic differential susceptibility normalized to its static value ($\chi /\chi_{0}$) is depicted in Figure 2a, showing a good agreement with the findings presented in [13]. The final solution for the Young’s modulus is achieved by the superposition of the purely mechanical component ($E_{0}$) and the magnetically induced component (∆E). When taking into account a hard axis magnetization process, it yields:

(10) $$E_{f}=\left\{\begin{array}{cc} \begin{array}{c} {\left(\frac{1}{E_{0}}+\frac{1}{∆E}\right)}^{-1}\\ E_{0} \end{array} & \begin{array}{c} \left|H\right|<H_{K}\\ \left|H\right|>H_{K} \end{array} \end{array}\right.$$

With the material parameters provided in Appendix A, we assessed the changes in the Young’s modulus of FeCoSiB with respect to the applied magnetic field, denoted as the Δ*E* effect (solid curve in Figure 2b). The magnetic field values are normalized to the effective anisotropy field $H_{K}$. As the magnetic field strength is increased, the Young’s modulus exhibits a non-linear decrease, reaching a minimum of approximately 95.7 GPa at the critical magnetic field of $\left|H\right|=H_{K}$. However, when the magnetic field surpasses the critical value of $H_{K}$, the Young’s modulus adopts the value of its purely mechanical component $E_{0}$ = 150 GPa.

To provide a means of comparison, we overlaid the phase shift measurement results obtained from an SAW delay line that incorporates an FeCoSiB layer with identical material parameters to those employed in our calculations (dashed curve in Figure 2b). To acquire the phase shift depicted in Figure 2b, the sample was characterized in a magnetically, electrically, and acoustically shielded chamber inside a solenoid. The phase is analyzed as a function of the DC bias magnetic field at a synchronous frequency of 251.3 MHz and a power of 1 dBm. Further details regarding the fabrication and measurement methods can be found in [31].

The magnetostrictive material parameters utilized in our study are acquired for a continuous layer of FeCoSiB. It is important to note that these parameters would be slightly different for FeCoSiB pillars of the same thickness. To construct a more precise model, one has to take the geometry of the magnetostrictive material into consideration and use the material parameters specific to the FeCoSiB pillars in simulations. To achieve this, one would need to fabricate pillars of approximately the desired size and conduct measurements to acquire the material parameters corresponding to these fabricated pillars. Subsequently, the obtained parameters can be employed for calculating a more accurate susceptibility and Young’s modulus values, which would then serve as the basis for fine-tuning the design of the PnC.

The overall sensitivity of an SAW magnetic field sensor, which will be further elaborated in subsequent sections, can be expressed as follows [9]:

(11) $$S=\frac{\partial \varphi }{\partial H}=\frac{\partial E_{f}}{\partial H}\cdot \frac{\partial v}{\partial E_{f}}\cdot \frac{\partial \varphi }{\partial v}=S_{mag}\cdot S_{str}\cdot S_{geo},$$

where $S_{mag}=\partial E_{f}/\partial H$, i.e., the magnetic sensitivity of the magnetostrictive material, is associated with the variation in the Young’s modulus of the magnetostrictive material $E_{f}$ when subjected to an external magnetic field H. Based on the data presented in Figure 2b, the Young’s modulus exhibits nearly linear changes within the interval of $0.6H_{K}<\left|H\right|<0.9H_{K}$, yielding a magnetic sensitivity of $S_{mag}$~−31.9 GPa/mT.

The change in the SAW velocity with respect to variations in the Young’s modulus is quantified by structural sensitivity $S_{str}=\partial v/\partial E_{f}$. This sensitivity value depends on the structure of the delay line through which the SAW propagates. It has been demonstrated, for instance, that increasing the thickness of the guiding layer beneath the magnetostrictive material in a delay line configuration can result in higher values of structural sensitivity [9]. In the following sections, we will show that an improved structural sensitivity can be achieved by replacing the magnetostrictive layer with a periodic arrangement of magnetostrictive pillars, forming a PnC.

A change in SAW velocity at a given frequency *f* induces a phase shift at the end of the delay line with a length *l*. One can measure this phase shift to determine the external magnetic field strength. The quantity representing the degree of phase shift per change in wave velocity is known as the geometric sensitivity $S_{geo}=\partial \varphi /\partial v$. The overall sensitivity is determined by multiplying these three sensitivity values, as previously mentioned.

## 3. Results

PnCs are the acoustic counterparts to atomic lattices in solid-state physics [39] or photonic crystals in optics [40,41]. They are designed to manipulate the acoustic waves in fluids or elastic waves in solids. They exhibit stop bands within their transmission spectra, effectively blocking the propagation of acoustic waves either in a particular direction or in all directions [18,42,43,44,45]. The precise location and width of the acoustic bandgaps are determined by factors such as the lattice structure, inclusion shapes, and constituent materials. In addition to bandgaps, PnCs exhibit fascinating mode dispersion characteristics, giving rise to peculiar phenomena such as slow wave propagation [46,47] or the formation of localized modes [48,49]. By exploiting these phenomena, one can efficiently filter, confine, or steer the acoustic waves in a desired fashion [50,51,52,53,54,55]. In the following section, we calculate the band structure and demonstrate the dispersion of our proposed PnC.

### 3.1. Band Structure of the PnC

As depicted in Figure 1, we consider a 2D square arrangement of FeCoSiB pillars on the surface of a thick silicon substrate covered by a 1 μm thick piezoelectric layer (AlScN) and subsequently a 4.5 μm thick SiO_2_ layer. A unit cell of the PnC is displayed with more details in the magnified view of Figure 1. The lattice constant of the PnC is denoted as *a*, while the radius and thickness of the pillars are represented by *r_p_* and *h_p_*.

We employ COMSOL Multiphysics 6.1^®^ to compute the band structure of the PnC. In this approach, we limit our calculations to a single unit cell of the PnC. To mimic infinite periodicities in both the *x* and *y* directions, we apply Bloch–Floquet conditions at the boundaries of the unit cell [18,44]. By altering the wave vector within the first Brillouin zone and solving an eigenvalue problem, one can determine the eigenfrequencies associated with each wave vector. The acquired eigenfrequencies signify the allowed acoustic modes that can propagate through the PnC.

**Figure 3:**
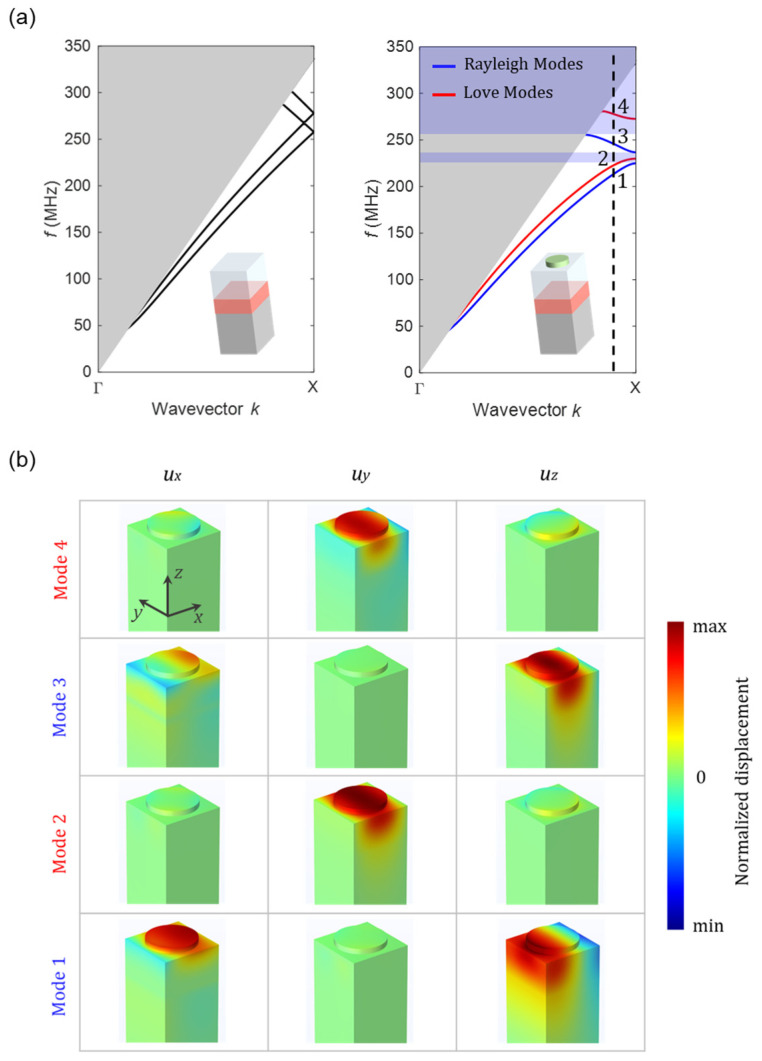
(**a**) Band structure of (right panel) the proposed PnC with *a* = 8 μm, *r_p_* = 2.8 μm, and *h_p_* = 600 nm, (left panel) a similar configuration without the PnC structure. Both are calculated in the Γ-Χ direction of the first Brillouin zone. The insets provide schematic representations of the corresponding configurations. Rayleigh and Love modes are indicated by blue and red lines, respectively. The grey- and purple-shaded areas outline the sound cone and the stop bands for the Rayleigh modes. (**b**) Displacements of the first four modes, decomposed into the three components *u_x_*, *u_y_*, and *u_z_*, calculated at a certain wave vector *k_x_* close to the point X of the first Brillouin zone, as indicated by a dashed line in (**a**).

The band structure calculated in the Γ-Χ direction for a PnC with *a* = 8 μm, *r_p_* = 2.8 μm, and *h_p_* = 600 nm is presented in the right panel of Figure 3a, wherein Rayleigh and Love modes are indicated by blue and red lines, respectively. The dispersion curve reveals the presence of two bandgaps (indicated by purple-shaded areas) where the propagation of Rayleigh modes in the *x*-direction is prohibited. However, a Rayleigh mode is evident within the frequency range between the two bandgaps (*f* = 237–257 MHz). This observation confirms that the Rayleigh mode launched by the IDT, with the center frequency of *f*~250 MHz, is permitted to propagate through the PnC.

The left panel of Figure 3a illustrates the band structure of a comparable configuration without the PnC structure. In this case, each band exhibits a nearly constant slope, indicating a constant group velocity, across various wave vectors. It is evident that the two Rayleigh modes, similar to the two Love modes, show degeneracy at the edge of the Brillouin zone (at the Χ point), resulting in the closure of the bandgaps. The grey-shaded area on the band structure represents the sound cone. Acoustic waves that are confined to the surface and are guided through the PnC only exist in the areas of the band structure that fall outside the sound cone. Other modes emerge at frequencies that fall within the sound cone, resulting in their radiation into the bulk material.

In Figure 3b, we demonstrate the modal displacements of the first four bands at a wave vector close to the X point of the first Brillouin zone, as indicated by a dashed line in the right panel of Figure 3a. While eigenmodes 1 and 3 exhibit mostly sagittal (*u_x_*,*u_z_*) polarizations with negligible *u_y_* values, the *y*-component of the displacement vector is dominant in eigenmodes 2 and 4. The former correspond to Rayleigh waves, whereas the latter signify shear horizontal surface waves, namely Love waves. Note that consistent scaling has been applied to all displacements.

### 3.2. Transmission through PnC

**Figure 4:**
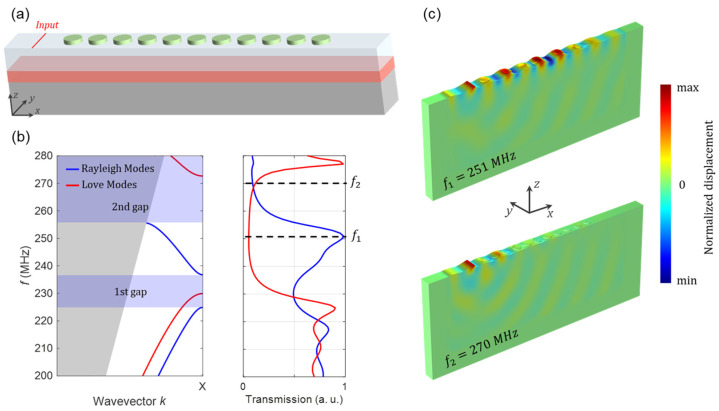
(**a**) The schematic illustration of the model utilized for transmission calculations. An SAW wave is triggered using a line source in front of a PnC composed of 11 FeCoSiB pillars along the *x*-direction. A periodic boundary condition is applied along the *y*-direction to consider an infinite periodic structure and a line source. PMLs are used in the *x*- and *z*-directions to prevent reflections from the domain boundaries. (**b**) Transmission spectra of Rayleigh (blue) and Love (red) modes acquired by calculating average displacements along a line at the end of the PnC. (**c**) Displacement of Rayleigh waves stimulated by the line source at *f*_1_ = 251 MHz (above) and *f*_2_ = 270 MHz (below). The same scaling is applied to both displacements.

To study the transmission of the SAWs through the PnC, we employ the model depicted in Figure 4a. In this configuration, an SAW wave with a specific polarization (*u_x_*, *u_y_, u_z_*) is stimulated by applying a line source at one side of a PnC with finite number of periodicities along the *x*-direction (*n* = 11). The transmission spectra are then obtained via calculating the average displacements along a line at the opposite side of the PnC as a function of frequency. We implement a periodic boundary condition in the *y*-direction to simulate infinite periodic repetitions and an infinitely long line source. To prevent undesired reflections from the domain boundaries, we impose perfectly matched layers (PMLs) [56] in both the *x* and *z*-directions.

One can manually set the line source to exhibit two distinct polarizations: (i) (*u_x_*,*u_z_*) displacements capable of exciting sagittally polarized waves such as Rayleigh modes, or (ii) *u_y_* displacements serving as a source for shear horizontally polarized waves, e.g., Love modes. In each of the aforementioned cases, we compute the average of all displacement components (|*u_x_*| + |*u_y_*| + |*u_z_*|) along a line at the opposite side of the PnC as a function of the frequency. In Figure 4b, the acquired transmission spectra are depicted alongside the band structure of the PnC within the same frequency range for a better comparison. The red curve in the transmission spectra is calculated for a shear horizontally polarized line source and, therefore, corresponds to Love modes. In the frequency range of 230–273 MHz, corresponding to the bandgap of the Love modes, the transmission spectrum reveals minimal values. Within this bandgap, Love waves experience significant attenuation as they traverse the PnC. On the other hand, the blue curve is derived from a sagittally polarized line source, corresponding to Rayleigh modes. The transmission spectrum of the Rayleigh modes demonstrates two attenuation frequency regions, matching the first and second bandgaps in the band structure, marked by purple shading.

For a deeper exploration of Rayleigh wave propagation through the PnC, Figure 4c shows the displacement of Rayleigh waves excited by the line source at two distinct frequencies: *f*_1_ = 251 MHz, where maximum transmission occurs, and *f*_2_ = 270 MHz, located within the second bandgap of the Rayleigh modes. In the former case, a Rayleigh wave travels through the PnC structure, yielding a peak transmission due to resonant interactions with periodic structures. Interestingly, at the latter frequency, the PnC efficiently hinders the Rayleigh wave, resulting in minimal transmission.

**Figure 5:**
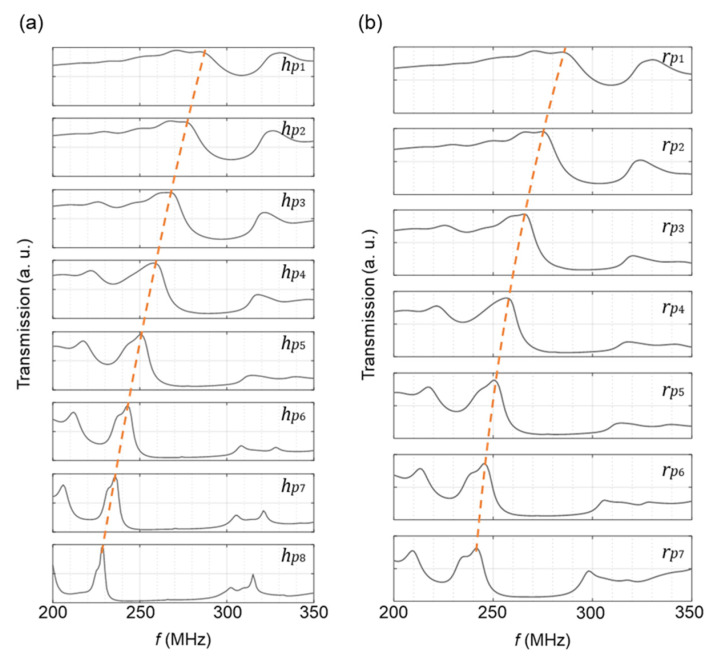
Transmission spectra of Rayleigh modes through a PnC with (**a**) *a* = 8 μm, *r_p_* = 0.35*a* = 2.8 μm and various thicknesses of the FeCoSiB pillars *h_p_*_1_ = 200 nm, *h_p_*_2_ = 300 nm, *h_p_*_3_ = 400 nm, *h_p_*_4_ = 500 nm, *h_p_*_5_ = 600 nm, *h_p_*_6_ = 700 nm, *h_p_*_7_ = 800 nm, and *h_p_*_8_ = 900 nm, and (**b**) *a* = 8 μm, *h_p_* = 600 nm and various radii of the FeCoSiB pillars *r_p_*_1_ = 0.15*a*, *r_p_*_2_ = 0.2*a*, *r_p_*_3_ = 0.25*a*, *r_p_*_4_ = 0.3*a*, *r_p_*_5_ = 0.35*a*, *r_p_*_6_ = 0.4*a*, and *r_p_*_7_ = 0.45*a*.

The transmission peak of surface acoustic modes propagating through the PnC can be finely tuned by modifying the dimensions of the pillars. This feature allows for the design of PnCs that resonate at desired frequencies. In this context, we investigate the impact of the thickness (Figure 5a) and radius (Figure 5b) of FeCoSiB pillars on the resonance frequency of the PnC, while the periodicity is kept constant (*a* = 8 μm). At a fixed radius (*r_p_* = 0.35*a* = 2.8 μm), increasing the thickness of the pillars (*h_p_*_1_ = 200 nm, *h_p_*_2_ = 300 nm, *h_p_*_3_ = 400 nm, *h_p_*_4_ = 500 nm, *h_p_*_5_ = 600 nm, *h_p_*_6_ = 700 nm, *h_p_*_7_ = 800 nm, and *h_p_*_8_ = 900 nm) results in sharper transmission peaks at lower frequencies. Furthermore, with an increase in the thicknesses of the pillars, a noticeable widening of both the first and second bandgaps is observed. An almost similar trend is evident when increasing the pillar radius (*r_p_*_1_ = 0.15*a*, *r_p_*_2_ = 0.2*a*, *r_p_*_3_ = 0.25*a*, *r_p_*_4_ = 0.3*a*, *r_p_*_5_ = 0.35*a*, *r_p_*_6_ = 0.4*a*, and *r_p_*_7_ = 0.45*a*) at a fixed thickness of *h_p_* = 600 nm. To ensure that the Rayleigh mode initiated by the IDT can propagate through the PnC with minimal dissipation, it is crucial to match the resonance frequency of the PnC with that of the Rayleigh mode initiated by the IDT, i.e., *f* ~ 250 MHz. To fulfill this requirement, we select pillar dimensions of *r_p_* = 0.35*a* = 2.8 μm and *h_p_* = 600 nm.

**Figure 6:**
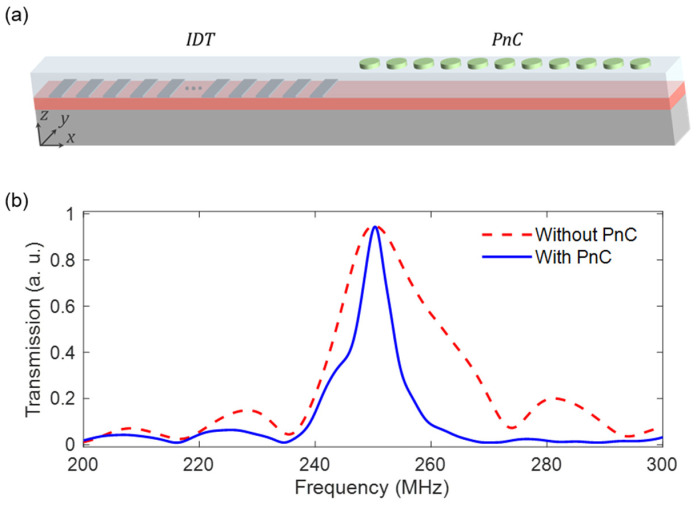
(**a**) Transmission model with IDT and PnC. The IDT consists of 12 pairs of Aluminum split-finger structures with a periodicity of 16 µm, a finger width of 4 µm, and a thickness of 150 nm, giving rise to a Rayleigh mode with the center frequency of *f*~250 MHz that propagates through the delay line in the *x*-direction. (**b**) Transmission spectra of the Rayleigh waves initiated by the IDT, computed at the end of the delay line without (red dashed curve) and with (blue solid curve) the PnC incorporated into the model.

To construct a more realistic model, we incorporate into the transmission setup an IDT comprising 12 pairs of Aluminum split-finger structures with a periodicity of 16 µm, a finger width of 4 µm, and a thickness of 150 nm, stimulating a Rayleigh mode with the center frequency of *f*~250 MHz. The complete simulation model is presented in Figure 6a. The Rayleigh wave initiated via the IDT traverses the patterned delay line, giving rise to a transmission spectrum characterized by a distinct and sharp peak, centered around the frequency *f*~250 MHz (blue solid curve in Figure 6b). In the absence of the PnC structure, on the other hand, the transmission spectrum exhibits a broader peak, as indicated by the red dashed curve in Figure 6b. This broadening of the transmission peak is attributed to the absence of resonance effects typically induced by the PnC structure. It is important to note that, in this case, the width of the transmission peak can be reduced by increasing the number of fingers in the IDT.

### 3.3. ΔE Effect

**Figure 7:**
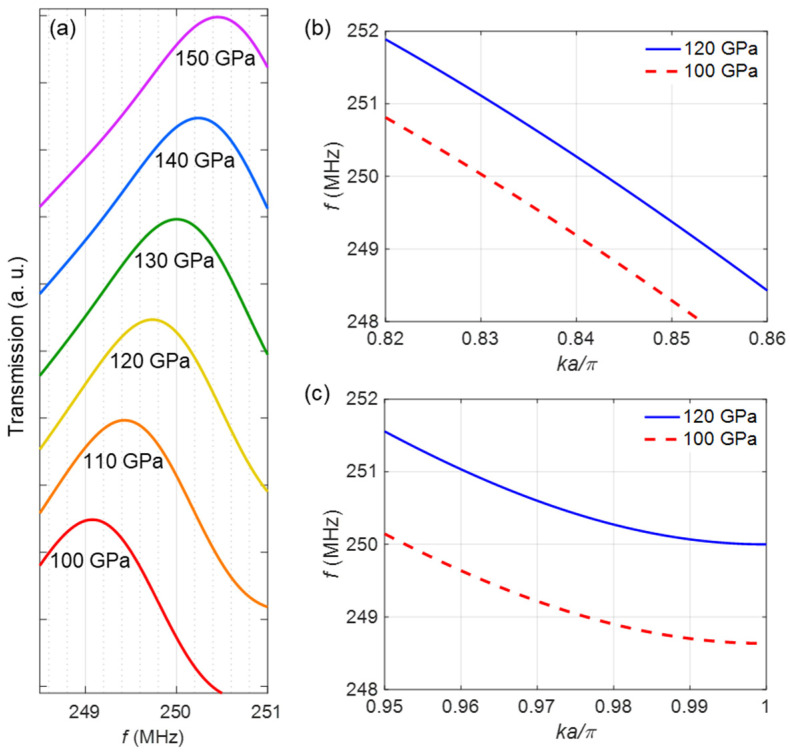
(**a**) Transmission spectra along the PnC with the Young’s modulus values varying in the range of 100–150 GPa. Band structure of the PnC with parameters (**b**) *a* = 8 μm, *r_p_* = 2.8 μm, and *h_p_* = 600 nm, and (**c**) *a* = 7 μm, *r_p_* = 2.45 μm, and *h_p_* = 700 nm, with the Young’s modulus values of 100 GPa (red dashed curve) and 120 GPa (blue solid curve). Only the second Rayleigh mode (mode 3 in Figure 3) is shown.

To explore the magnetic field sensing capability of our proposed device, we initially analyze the variations in the transmission peak frequency upon the application of an external magnetic field. In Figure 2b, we demonstrate that the application of varying magnetic fields results in observable changes in the Young’s modulus of FeCoSiB. Figure 7a displays the transmission spectra obtained for PnC structures comprising FeCoSiB pillars with varying Young’s modulus values. At $E_{f}$=150 GPa, representing the Young’s modulus of FeCoSiB in the absence of an external magnetic field, the transmission peak of the PnC occurs at the frequency of *f* = 250.5 MHz. By altering the Young’s modulus of the FeCoSiB pillars from 150 to 100 GPa through the application of a homogeneous magnetic field ranging from 0 to $H=0.9H_{K}$, the transmission peak of the PnC shifts slightly towards lower frequencies. It is evident form the figure that the transmission peak reaches a minimum frequency of *f*~249 MHz when $E_{f}$ = 100 GPa, corresponding to a homogeneous external magnetic field of $H=0.9H_{K}$. These frequency shifts can be detected through changes either in the amplitude or phase of the transmitted signal. In the former scenario, the transmission amplitude is acquired for various Young’s modulus values at a specific frequency, namely *f* = 250 MHz. The amplitude of the transmitted signal is determined by the Young’s modulus value and can therefore be correlated with the intensity of the applied magnetic field. 

In the latter case, however, the phase of the transmitted signal is analyzed for different Young’s modulus values at the given frequency of *f* = 250 MHz. The observed phase shift is connected to variations in the Young’s modulus, and consequently, to the applied magnetic field. In this study, we particularly concentrate on the latter scenario to determine the sensitivity of the magnetic field sensor. To quantify the structural sensitivity of the sensor, denoted as $S_{str}$, we evaluate the change in the SAW phase velocity (∆v) when the Young’s modulus of FeCoSiB is varied within the range of $100\;\mathrm{Gpa}<E_{f}<120\;\mathrm{Gpa}$. These values correspond to the applied magnetic field range of $0.6H_{K}<\left|H\right|<0.9H_{K}.$ Within this specified range, we observed maximal and nearly linear changes in the Young’s modulus in response to variations in the magnetic field. We calculated the band structures of PnCs composed of FeCoSiB pillars with two distinct Young’s modulus values: 100 GPa and 120 GPa. Figure 7b specifically exhibits the band diagram of the second Rayleigh mode (mode 3 in Figure 3) within a narrow frequency range centered around *f*~250 MHz. At the fixed frequency of *f* = 250 MHz, a decrease in the Young’s modulus from 120 GPa to 100 GPa leads to a reduction in the phase velocity, defined as v=2πf/k, from 3458 m/s to 3420 m/s. This yields a structural sensitivity equal to $S_{str}\sim 1.88\;\frac{m/s}{GPa}$.

To improve the structural sensitivity, one can fine-tune the PnC geometry so that the second Rayleigh band (mode 3 in Figure 3) intersects with the edge of the first Brillouin zone at *f* = 250 MHz. Close to the edge of the first Brillouin zone, the band exhibits an almost flat profile, resembling an SAW with a relatively small group velocity. As a result, even a small change in the Young’s modulus in this region leads to a large alteration in the phase velocity and, consequently, a noticeable phase shift. To demonstrate this, we present the band diagram of a similar PnC with parameters *a* = 7 μm, *r_p_* = 2.45 μm, and *h_p_* = 700 nm, centered around the frequency of *f* = 250 MHz, as shown in Figure 7c. With the same reduction in the Young’s modulus at *f* = 250 MHz, the phase velocity decreases from 3517 m/s to 3362 m/s, resulting in a structural sensitivity of approximately $S_{str}\sim 7.75\;\frac{m/s}{GPa}$.

The phase of an SAW with a given frequency *f* and phase velocity *v* at the end of a delay line with a length *l* is expressed as $\varphi =2\pi f\frac{l}{v}$. The geometric sensitivity is then defined as $S_{geo}=\partial \varphi /\partial v=-2\pi f\frac{l}{v^{2}}$. In our specific case, where the delay line consists of 11 pillars with a total length of *l* = 77 μm, we achieve a geometric sensitivity of $S_{geo}\sim -0.56\frac{{}^{\circ}s}{m}$ at the frequency of *f* = 250 MHz and the phase velocity of *v* = 3517 m/s. The overall sensitivity, which quantifies how responsive the phase of the SAW is to changes in the external magnetic field, can be obtained by multiplying the three contributions, i.e., the magnetic, structural, and geometric sensitivities. In this study, an overall sensitivity of $S\sim 138\;\frac{{}^{\circ}}{mT}$ is obtained. To provide a meaningful comparison with previous studies on SAW magnetic field sensors, we scaled the sensitivity reported in [9] to match the delay line length in our study, which is set at *l* = 77 µm. As a result, the normalized overall sensitivity achieved in our research is approximately 15 times greater than what was reported by Kittmann et al. [9].

Furthermore, we conducted a comparative analysis between the sensitivity of our proposed device and that of a similar device where the PnC was replaced by an unpatterned magnetostrictive layer with identical material properties. Our simulation findings indicate that, when the Young’s modulus of the FeCoSiB layer is reduced from 120 GPa to 100 GPa at *f* = 250 MHz, the phase velocity of the SAW traveling through the delay line decreases from 3146 m/s to 3077 m/s. This results in a structural sensitivity of $S_{str}\sim 3.45\;\frac{m/s}{GPa}$, a geometric sensitivity of $S_{geo}\sim -0.7\frac{{}^{\circ}s}{m}$, and an overall sensitivity of $S\sim 77\;\frac{{}^{\circ}}{mT}$.

## 4. Conclusions

In this study, we investigated the integration of a precisely engineered PnC structure into an SAW-based magnetic field sensor. Our exploration focused on improving the sensing capabilities by leveraging the potential of PnCs to manipulate acoustic wave propagation. By tailoring the dimensions and arrangement of the FeCoSiB pillars within the PnC, we achieved resonant modes that aligned with the surface acoustic modes generated by the IDTs. This alignment led to an enhanced interaction between the SAW and the magnetostrictive material, resulting in an improved sensor’s sensitivity.

Furthermore, we examined the Δ*E* effect, i.e., the effect of a change in the Young’s modulus of the magnetostrictive material due to an external magnetic field on SAW propagation. Our simulations highlighted the sensor’s responsiveness to variations in magnetic field, as evidenced by observable changes in the SAW phase velocity and consequently phase shifts in the output signal. The proposed sensor configuration offers a higher sensitivity compared to previously introduced SAW magnetic field sensors, making it a promising candidate for various practical applications.

In conclusion, our study highlights the potential of integrating PnCs into SAW-based magnetic field sensors with the aim of improving sensing functionality. As the demand for accurate magnetic field detection grows across various industries, our approach offers a pathway towards more effective and reliable magnetic field sensing solutions.

## Data Availability

The data that support the findings of this study are available from the corresponding authors upon reasonable request.

## Appendix A. Material Parameters

As the substrate, we use isotropic silicon with a Young’s modulus of E_Si_ = 169 GPa, a Poisson’s ratio of *υ*_Si_ = 0.28, and a mass density of *ρ*_Si_ = 2329 kg/m^3^ [38,57].

Al_0.68_Sc_0.32_N is exploited as the piezoelectric material, whose material parameters are as follows [58]:

$$C_{AlScN}=\left(\begin{array}{cc} \begin{array}{ccc} \begin{array}{c} \begin{array}{c} 307\\ 133\\ 123 \end{array}\\ \begin{array}{c} 0\\ 0\\ 0 \end{array} \end{array} & \begin{array}{c} \begin{array}{c} 133\\ 307\\ 123 \end{array}\\ \begin{array}{c} 0\\ 0\\ 0 \end{array} \end{array} & \begin{array}{c} \begin{array}{c} 123\\ 123\\ 230 \end{array}\\ \begin{array}{c} 0\\ 0\\ 0 \end{array} \end{array} \end{array} & \begin{array}{ccc} \begin{array}{c} \begin{array}{c} 0\\ 0\\ 0 \end{array}\\ \begin{array}{c} 110\\ 0\\ 0 \end{array} \end{array} & \begin{array}{c} \begin{array}{c} 0\\ 0\\ 0 \end{array}\\ \begin{array}{c} 0\\ 110\\ 0 \end{array} \end{array} & \begin{array}{c} \begin{array}{c} 0\\ 0\\ 0 \end{array}\\ \begin{array}{c} 0\\ 0\\ 87 \end{array} \end{array} \end{array} \end{array}\right)\;\mathrm{GPa},$$

$$\rho_{AlScN}=3320\;\mathrm{kg}/m^{3},$$

$$e_{AlScN}=\left(\begin{array}{ccc} \begin{array}{cc} 0 & 0\\ 0 & 0\\ -0.69 & -0.69 \end{array} & \begin{array}{cc} 0 & 0\\ 0 & -0.23\\ 2.8 & 0 \end{array} & \begin{array}{cc} -0.23 & 0\\ 0 & 0\\ 0 & 0 \end{array} \end{array}\right)\;C/m^{2},$$

$${\varepsilon_{r}}_{AlScN}=\left(\begin{array}{ccc} 10 & 0 & 0\\ 0 & 10 & 0\\ 0 & 0 & 13 \end{array}\right),$$

$$\mu_{AlScN}=\left(\begin{array}{ccc} 0.4\pi & 0 & 0\\ 0 & 0.4\pi & 0\\ 0 & 0 & 0.4\pi \end{array}\right)\;µH/m,$$

As the magnetostrictive material, we consider isotropic FeCoSiB with a Young’s modulus of $E_{0}$= 150 GPa, a Poisson’s ratio of *υ* = 0.3, and a mass density of *ρ* = 7250 kg/m^3^ [16]. In our Δ*E* effect calculations, we set the first-order anisotropy constant to *K* = 1400 J·m^−3^, the static differential magnetic susceptibility to *χ*_0_ = 639, and the saturation magnetostriction to *λ*_s_ = 35 ppm. Furthermore, the saturation flux density is *μ*_0_*M*_s_ = 1.48 T.
